# P07 Rheumatoid arthritis, joint pain with raised CRP: more than a flare

**DOI:** 10.1093/rap/rkab068.006

**Published:** 2021-10-19

**Authors:** Eirini Giavri, Shoma Banerjee, Ioanna Papadaki, Bill Smith, Simon Bowman

**Affiliations:** Milton Keynes University Hospital NHS Foundation Trust Hospital, Milton Keynes, United Kingdom

## Abstract

**Case report - Introduction:**

Rheumatoid arthritis is a chronic inflammatory autoimmune systemic disorder of unknown aetiology that predominantly affects the joints. Moreover, it is a systemic illness associated with a variety of extraarticular manifestations, including haematological complications such as lymphoma. We are presenting a case highlighting the importance of surveillance in these patients, particularly elderly patients with longstanding disease.

**Case report - Case description:**

A 72-year-old female patient of African-Caribbean origin was referred to the rheumatology department from the community clinic for RA deterioration. She was diagnosed with RA RF anti-CCP positive in 2007 and was treated with sc MTX 25 mg weekly, HQ 200 mg bd, pred 5 mg and naproxen 250 mg. Her past medical history included HTN, OA, R TKR and degenerative lower back pain.

Patient reported: ‘Doctor, I ‘ve had RA for many years, but never before I had such a pain’.

She was complaining of multiple joint pains, morning stiffness of up to 2hrs, widespread pain and fatigue. She reported weight loss of 5 pounds in the last 2 months. For the last 2 weeks, she was suffering from lower back pain, L leg sciatica, with pins and needles, L leg weakness and incontinence. She was otherwise well.

On examination she had 8 mildly swollen, 0 tender joints, VAS score was 90/100. DAS-28 was 4.92.

Blood tests showed normocytic anaemia Hb 107, raised ESR 58 and CRP  124, previously 22 and 5.8. Anaemia screening, immunoglobulins and free light chains were normal. Biologic screening was normal.

Repeating the blood tests following IM steroid injection, ESR was 81 and CRP 187. Alternative diagnosis such as infection and malignancy were suspected. A plan was made to review in the clinic and arrange further investigations with a CT scan.

MRI spine was arranged which identified multiple osseous deposits L3-L5, T8-T12, T1, C2, retroperitoneal psoas mass, paravertebral mass and multiple retroperitoneal lymph nodes.

CT revealed lung nodules and splenic lesions possible metastatic and right ileac destructive lesion.

PET CT showed multiple active uptake in lymph nodes above and below diaphragm, the spleen and lung nodules, axial and appendicular skeleton.

Patient had a bone marrow biopsy which revealed diffuse large B-cell lymphoma.

**Case report - Discussion:**

This is a case of a patient with a 13-year history of rheumatoid arthritis, who was stable until last year and presented with worsening joint pain. Joint examination did not correlate with the severity of her pain. However. fluctuations in disease activity and variation throughout the day are common in rheumatoid arthritis and patient reported morning stiffness.  Differential diagnosis initially included RA flare up with the possibility that osteoarthritis, fibromyalgia and degenerative spinal disease could also exacerbate her pain.

However, ESR and CRP were significantly raised disproportionally for the joint count. Moreover, she had systemic symptoms with weight loss and fatigue raising the question of an alternative diagnosis such as malignancy or infection. Patient did not have obvious symptoms or signs of infection, and baseline investigations, such as CXR and urine dipstick were normal and TB spot was negative. However, there was concern for an occult infection. Malignancy could be a potential diagnosis as the risk increases with age, and haematological malignancies, particularly lymphoma, have been associated with RA. Myeloma could be an alternative diagnosis, based on anaemia and back pain; however, myeloma screening came back normal.

Patient was diagnosed with stage IVB diffuse large B-cell lymphoma with metastatic bone disease, paravertebral mass, retroperitoneal lymph nodes, psoas mass, pulmonary nodules, and splenic lesions. Her joint and back pain were related to metastatic bone disease. She was treated with 2 cycles R-CHOP, 4 cycles R mini-CHOP. She repeated the PET CT which showed improvement. CRP dropped to 1.3.

**Case report - Key learning points:**

Rheumatoid arthritis is a systemic disease and the raised inflammatory markers do not necessarily indicate RA flare. We should consider other causes in our differential diagnosis, such as infection and malignancy.

Studies have shown 2-fold increased risk for lymphoma in RA patients, HL, NHL and particularly the diffuse large B-cell Lymphoma. The risk of having lymphoma correlates with disease activity. DMARD treatment including anti-TNF does not seem to increase the risk which is probably driven by the systemic inflammation causing persistent immunologic stimulation, B cell clonal expansion and transformation along with decreased T suppressor cells and NK activity.

Therefore, EULAR recommends systemic screening for infections and malignancy, along with other co-morbidities as part of the routine care in patients with rheumatoid arthritis. At the end, we should always listen to the patient’s story.

